# Cry1Ac toxin binding in the velvetbean caterpillar *Anticarsia gemmatalis*: study of midgut aminopeptidases N

**DOI:** 10.3389/fphys.2024.1484489

**Published:** 2024-10-29

**Authors:** M. D. Lanzaro, I. Padilha, L. F. C. Ramos, A. P. G. Mendez, A. Menezes, Y. M. Silva, M. R. Martins, M. Junqueira, F. C. S. Nogueira, C. D. AnoBom, G. M. Dias, F. M. Gomes, D. M. P. Oliveira

**Affiliations:** ^1^ Departamento de Bioquímica, Instituto de Química, Universidade Federal do Rio de Janeiro, Rio de Janeiro, Brazil; ^2^ Instituto de Biofísica Carlos Chagas Filho, Universidade Federal do Rio de Janeiro, Rio de Janeiro, Brazil; ^3^ Instituto Nacional de Entomologia Molecular, Rio de Janeiro, Brazil

**Keywords:** aminopeptidase N, Cry toxin, receptor, *Bacillus* thuringiensis, *Anticarsia gemmatalis*

## Abstract

The velvetbean caterpillar *Anticarsia gemmatalis* is one of the main soybean defoliators in Brazil. Currently, the main biopesticide used to control insect pests worldwide is the bacteria *Bacillus thuringiensis* (Bt), which produces entomopathogenic Crystal toxins (Cry) that act in the midgut of susceptible insects, leading them to death. The mode of action of Cry toxins in the midgut involves binding to specific receptors present on the brush border of epithelial cells such as aminopeptidase N (APN), alkaline phosphatase (ALP), cadherin, and others. Mutations in these receptors, among other factors, may be involved in the development of resistance; identification of functional Cry receptors in the midgut of *A. gemmatalis* is crucial to develop effective strategies to overcome this possible scenario. This study’s goal is to characterize APNs of *A. gemmatalis* and identify a receptor for Cry1Ac in the midgut. The interaction of Bt spores with the midgut epithelium was observed *in situ* by immunohistochemistry and total aminopeptidase activity was estimated in brush border membrane vesicle (BBMV) samples, presenting higher activity in challenged individuals than in control ones. Ten APN sequences were found in a *A. gemmatalis*’ transcriptome and subjected to different *in silico* analysis, such as phylogenetic tree, multiple sequence alignment and identification of signal peptide, activity domains and GPI-anchor signal. BBMV proteins from 5th instar larvae were submitted to a ligand blotting using activated Cry1Ac toxin and a commercial anti-Cry polyclonal antibody; corresponding bands of proteins that showed binding to Cry toxin were excised from the SDS-PAGE gel and subjected to mass spectrometry analysis, which resulted in the identification of seven of those APNs. Quantitative PCR was realized to compare expression levels between individuals subjected to sublethal infection with Bt spores and control ones, presenting up- and downregulations upon Bt infection. From these results, we can infer that aminopeptidases N in *A. gemmatalis* could be involved in the mode of action of Cry toxins in its larval stage.

## 1 Introduction

*Bacillus thuringiensis* (Berliner, 1915; Bacillales: Bacillaceae) (Bt) is a gram-positive, spore-forming bacteria known for its entomopathogenic effect against susceptible insects (Bravo et al., 2007; Sanchis, 2011). During its sporulation phase of growth, it produces insecticidal proteins as crystal inclusions (Cry toxins) that are pathogenic to several insect models, including known insect pests from the orders Lepidoptera, Coleoptera, and Hymenoptera (Bravo et al., 2007). For this reason, these proteins have been used worldwide in insecticidal sprays and, in later years, incorporated in genetically engineered crops (Horikoshi et al., 2021b; Pozebon et al., 2020; Bernardi et al., 2012).

Soybean (*Glycine max* (L.) Merrill, 1917) is one of the major agricultural commodities worldwide, with great importance in the global market, with projections that indicate that the brazilian share of global trade could increase to over 60% until 2033 (dos Reis et al., 2020; Valdes et al., 2023). Brazil is currently the main producer and exporter of soybean (CONAB, 2023; FAO, 2024), with 154.6 million tons of grains produced in the 2022/23 harvest (CONAB, 2023). The velvetbean caterpillar *Anticarsia gemmatalis* (Hübner, 1818; Lepidoptera: Erebidae) is one of the soybean’s main defoliators in the Americas (Hoffman-Campo et al., 2000; Bernardi et al., 2012), causing great damage to the production of this grain. While chemical pesticides have typically been used in *A. gemmatalis* control, Bt-based biopesticides have been thoroughly used in the management of this species’ populations in the field (Knaak and Fiuza, 2005; Fernandes et al., 2021). In recent years, genetically modified soybean cultivars expressing insecticidal Cry proteins from *B. thuringiensis* (Bt crops) are quickly becoming a key tool in the management of pests, after its commercial availability (Pozebon et al., 2020; Bernardi et al., 2012; Chattopadhyay and Banerjee, 2018). Bt soybean MON 87701 × MON 89788 (Intacta RR2 PRO®) provides protection against *A. gemmatalis* in a high-dose manner, presenting high levels of Cry1Ac expression throughout the planting season conferring complete neonate mortality and effectively managing their populations in the field (Tabashnik et al., 2023; Horikoshi et al., 2021a; Bernardi et al., 2012).

Once ingested, Bt crystals are solubilized in the insect midgut alkaline environment, originating Cry protoxins, which are further activated by midgut proteases. Upon activation, Cry toxins bind to specific receptors in the midgut brush border membranes of epithelial cells and undergo conformational changes that cause the formation of oligomers, which are then inserted into the cell membrane. Cry toxin oligomerization at the plasma membrane forms pores, which leads to osmotic imbalance and cell lysis, which ultimately leads to insect death (Bravo et al., 2005; Adang et al., 2014; Palma et al., 2014; Melo et al., 2014; Liu et al., 2021). Four classes of proteins seem to be the major gut receptors for Cry toxins: aminopeptidase N (APN), alkaline phosphatase (ALP), cadherin (CAD), and ATP binding cassette subfamily C member 2 (ABCC2) transporters (Endo, 2022; Soberón et al., 2018; Bravo et al., 2004; Bravo et al., 2007; Gómez et al., 2002; Gómez et al., 2006; Pacheco et al., 2009). After activation, Cry toxins bind to APNs, abundant in lipid rafts in the membrane, which promotes the localization and concentration of activated toxins in these regions (Xu et al., 2014; Liu et al., 2021). The abundance of APN and its lower affinity for Cry toxins compared to receptors like CAD may allow it to act as a “toxin sink” that concentrates Cry proteins at the midgut membrane surface. APN can then pass the toxin to other receptors while also facilitating insertion of the oligomeric pore complex into the membrane. Accordingly, a decrease in total aminopeptidase activity was observed in strains resistant to Cry toxins (Yang et al., 2010). The APN (EC.3.4.11.2) consists of a class of metalloproteases that act in the midgut brush border of insect larvae cleaving N-terminal amino acids from peptides during digestion (Adang, 2004). Knight et al. (1994) first identified an APN as a Cry toxin-binding protein and putative receptor in *Manduca sexta* and since then several other works have identified these proteins as Cry receptors in different insect species (Adang, 2004; Pigott and Ellar, 2007; Liu et al., 2021). APNs present features such as aminopeptidase motif “GAMENWG,” Zn^++^-binding motif “HEXXHX18E,” a signal peptide in the N-terminal end and GPI-anchor peptide in the C-terminal end, which facilitates their attachment to the brush border, along with several O- and N-glycosylation sites (Pigott and Ellar, 2007; Adang, 2004). Based on their amino acid sequence similarity, they are classified into 13 clusters in Lepidoptera, with several isoforms expressed in the midgut (Guo et al., 2020; Crava et al., 2010; Hughes, 2014; Lin et al., 2014). The identification of lepidopteran APNs has allowed studies of the role of these proteins in the mode of action of Cry toxins, demonstrating their involvement in the pathogenesis of Bt toxins, mainly as functional receptors for these toxins (Gill and Ellar, 2002; Rajagopal et al., 2003; Wang et al., 2005).

Despite the importance of the velvetbean caterpillar (*A. gemmatalis*) as a major soybean pest, limited research has been conducted on the functional receptors for Cry toxins in this species. Previous studies have demonstrated the binding of various Cry toxins to *A. gemmatalis*’ midgut brush border membrane vesicles (BBMV) through competition-binding assays (Bel et al., 2017; Bel et al., 2019) and to the midgut epithelial tissue using biotinylated toxins (Fiuza et al., 2013). Additionally, a membrane-associated alkaline phosphatase (ALP) characterized in the midgut of *A. gemmatalis* showed interaction with Cry1Ac toxin *in vitro* through enzyme-linked immunosorbent assay (da Silva et al., 2019), suggesting its potential role as a Cry toxin ligand. Even though APNs are commonly described and studied as potential Cry receptors in other lepidopteran species, no works described these proteins in *A. gemmatalis*. In this study, we characterized *A. gemmatalis*’ aminopeptidases (AgAPNs) and identified those that positively bound to Cry toxin *in vitro*; analyses of AgAPN gene expression after Bt spore feeding bioassays demonstrated that some of these proteins could be involved in Cry’s mode of action, due to changes in expression in this condition. These findings will contribute to future works, aiding the understanding of potential APN role in Cry toxin mode of action in *A. gemmatalis*.

## 2 Materials and methods

### 2.1 Insects

A colony of *A. gemmatalis* was established in the lab using eggs obtained from EMBRAPA SOJA, Londrina, PR, Brazil. Larvae were reared on an artificial diet previously described by Hoffmann-Campo et al. (1985) and maintained under 25°C ± 3°C, 70% ± 10% humidity, and 14:10 h (light/dark) photoperiod.

### 2.2 Immunohistochemistry

For the immunohistochemistry assay, 0.5 mg/mL of Bt spores was mixed in the artificial diet fed to 4th-5th instar larvae; the control group was fed in an artificial diet mixed with distilled water. Samples were obtained after 12 h of Bt exposure and controls without exposure to the spores were also monitored under the same conditions. Challenged and control larvae were dissected and the gut of each was washed in phosphate-buffered saline [PBS; 137 mM NaCl, 2.7 mM KCl, 10 mM phosphate (pH 7.4)] to remove any unbound material before sample fixation. Tissues were fixed in 4% formaldehyde, 0.1% glutaraldehyde in 0.1 M sodium cacodylate buffer (pH 7.2) (Gomes et al., 2013) and stored in 4°C until use, for no longer than a week. After fixation, tissues were washed in 0.1 M sodium cacodylate buffer and incubated in blocking buffer (2% bovine serum albumin, 0.3% Triton X-100, PBS) for 2 h, followed by incubation in 1:250 commercial polyclonal primary anti-*B. thuringiensis* Cry1Ab Toxin antibody (Abcam Inc.; catalog number #ab51586) for 2 h in blocking buffer. After being washed in the blocking buffer, the tissues were incubated in 1:500 anti-rabbit Alexa-488-conjugated secondary antibody for 2 h, washed and incubated in 0.1 μg/mL DAPI. Whole gut samples were observed in a Zeiss 910 LSM confocal microscope.

### 2.3 *In silico* analysis of APN sequences

Raw RNA-Seq data of *A. gemmatalis* was obtained from a previously published study by Pezenti et al. (2021) (SRA accession number: PRJNA387150). The transcriptome was reassembled following the pipeline described in the original publication. APN amino acid sequences were identified through eggNOG-mapper (Cantalapiedra et al., 2021) and aligned using the Clustal W server (https://www.ebi.ac.uk/jdispatcher/msa/clustalo). For the phylogenetic tree, recovered APN sequences from nine lepidopteran species (*M. sexta*, *Heliothis virescens*, *Spodoptera frugiperda*, *Spodoptera litura*, *Helicoverpa armigera*, *Trichoplusia ni*, *Bombyx mori*, *Plutella xylostella*, and *Ostrinia furnacalis*), along with five sequences of *Homo sapiens*’ APNs to form an outgroup (Supplementary Table S1). Alignment was conducted with Clustal W on MEGA 11 (Tamura et al., 2021) and the phylogenetic analysis was performed in RAxML (Random Axelerated Maximum Likelihood) (Stamatakis, 2014) software using the maximum likelihood (ML) method with a bootstrapping procedure with 1,000 replicates. The tree was visualized by FigTree (http://tree.bio.ed.ac.uk/software/figtree/). The presence and location of signal peptide cleavage sites in the APN amino acid sequences were determined using the SignalP 5.0 Server (https://services.healthtech.dtu.dk/services/SignalP-5.0/). The presence of glycosylphosphatidylinositol (GPI) anchors was predicted using the PredGPI software (https://busca.biocomp.unibo.it/predgpi/). Potential N-linked and O-linked glycosylation sites were analyzed using the NetNGlyc1.0 (https://services.healthtech.dtu.dk/services/NetNGlyc-1.0/) and NetOGlyc4.0 (https://services.healthtech.dtu.dk/services/NetOGlyc-4.0/) programs, respectively. Schematic representations of the APN sequences, including the locations of signal peptide cleavage sites, GPI anchors, and glycosylation sites, were generated using the Illustrator for Biological Sequences (IBS, 2022) software (https://ibs.renlab.org/#/home).

### 2.4 Preparation of midgut brush border membrane vesicles (BBMVs)

Midguts from 5th instar larvae of *A. gemmatalis* were longitudinally dissected and washed in saline solution (NaCl 0.15 M). The samples were stored in ice-cold SET buffer (0.15 M sucrose, 17 mM tris[hydroxymethyl]aminomethane (Tris), 5 mM ethylene glycol-bis (β-aminoethyl ether)-N,N,N′,N′-tetraacetic acid (EGTA); pH 7.5) and kept at −20°C until use, for no longer than a week. BBMVs were prepared following the MgCl_2_ differential precipitation method described in Wolfersberger et al. (1987) with some modifications. Briefly, the dissected midgut-epithelium samples were homogenized in SET buffer with a glass potter, and an equal volume of 24 mM MgCl_2_ was added to the homogenate. Samples were incubated on ice for 15 min and then centrifuged at 3,000 *g* for 15 min at 4°C. The pellet was discarded, and the supernatant was centrifuged at 36,603 *g* for 30 min at 4°C. The pellet was resuspended in SET buffer and the centrifugation cycle was repeated. The final pellet was resuspended in 4-(2-hydroxyethyl) piperazine-1-ethane-sulfonic acid (HEPES) buffer (50 mM; pH 7.2). This resuspended pellet (P3) containing the proteins from BBMVs was quantified for total protein content using Pierce™ 660 nm Protein Assay (Thermo Scientific); bovine serum albumin (BSA; Sigma Chemical Company) was used as standard. The BBMV protein profile was observed by (12%) sodium dodecyl sulfate polyacrylamide gel electrophoresis (SDS-PAGE) (Laemmli, 1970).

### 2.5 Total leucine aminopeptidase activity in midgut BBMV samples

For total leucine aminopeptidase activity assay, a sublethal concentration of Bt spores (0.1323 mg/mL) was mixed in the artificial diet fed to 4th-5th instar larvae; the control group was fed in the artificial diet mixed with distilled water. This sublethal concentration was estimated in a bioassay that determined lethal concentrations of Bt spores to *A. gemmatalis* colony kept in the lab (data not shown). After 48 h of exposure to Bt, midguts were dissected and pooled (4 midguts per sample), with three biological replicates performed for the assay. Samples were then subjected to the preparation of BBMVs protocol and used in the total aminopeptidase activity assay. Total protein concentration was determined using the Pierce™ 660 nm Protein Assay Kit (Thermo Scientific) according to the manufacturer’s instructions, with bovine serum albumin (BSA) as the standard. Samples were diluted to a final concentration of 0.05 mg/mL in 0.1 M Tris-HCl buffer (pH 8.6) for the measurement of total aminopeptidase activity. Total aminopeptidase activity was assessed using the chromogenic substrate leucine-p-nitroanilide (LpNA) (Sigma, St. Louis, MO), as previously described by Valaitis et al. (1999) and Erlanger et al. (1961). Briefly, 5 µg of each diluted sample was mixed with 0.5 mM LpNA in 0.1 M Tris-HCl buffer (pH 8.6) in a microplate well. The enzymatic reaction was monitored by measuring the increase in optical absorbance at 405 nm using a SpectraMax® M2e microplate reader (Molecular Devices). Absorbance readings were taken every 30 s for a total of 15 min.

The mean velocity (Mean V) was calculated as an increase of absorbance per min in the linear portion of initial velocity of the enzymatic reaction using the GraphPad Prism 8.0. One unit of the enzyme activity was defined as the amount of enzyme that hydrolyzed 1 µmol of substrate to chromogenic product per min. Specific aminopeptidase activity calculations were performed based on the extinction coefficient (9.9 mM^−1^ cm^−1^) for p-nitroaniline (Hafkenscheid, 1984). APN activity was estimated from 3 reactions (replicates) for each condition (control and challenged) and sample type. Specific APN activities were presented as means and standard errors of the mean (SEM). Two-way ANOVA was used to determine differences in APN activity between the two conditions (control and challenged) for each sample.

### 2.6 Cry toxin purification and activation

*B. thuringiensis* subesp. *kurstaki* LFB-FIOCRUZ 475 (Bt) was kindly provided by Leon Rabinovitch (CCGB/IOC/Fiocruz/MS). This strain was used to produce Cry toxins, following Coleção de Culturas do Gênero *Bacillus* e Gêneros Correlatos (CCGB, Fundação Oswaldo Cruz) instructions. Bt spores were grown during 48 h in Nutrient Broth medium (Himedia) at 200 rpm at 37°C. This pre-inoculum was transferred to a new volume of nutrient broth and was shaken in the same growth conditions for 5 days until complete sporulation, as recommended instruction. After that, the culture was centrifuged at 7,500 *g* for 30 min at 4°C and the pellet was collected as spores/crystals-enriched fraction. Crystalline inclusions were solubilized by incubating the pellet in a 50 mM sodium carbonate buffer (pH 9.6) containing 0.1% 2-mercaptoethanol (SC buffer) under agitation for 2 h at room temperature. The mixture was centrifuged at 16,000 *g* for 30 min at 4°C. The supernatant was labeled as whole Cry1Ac toxin (WCT; about 130 kDa) and was either stored or further activated. WCT activation was performed by overnight incubation at 4°C with bovine pancreas trypsin (2 mg/mL). Following incubation, activated WCT was concentrated in an Amicon 30,000 MWCO spin filter for the removal of reminiscent trypsin. The molecular weight and the toxin integrity were checked by 10% SDS-PAGE (data not shown). This sample was named Cry1Ac toxin and used in the toxin overlay assay described below.

### 2.7 Toxin overlay assay in midgut BBMV proteins

The midgut BBMV proteins (50 µg) were separated by 12% SDS-PAGE (Laemmli, 1970), transferred to a nitrocellulose membrane, and Ponceau S was used to confirm protein transfer (Towbin et al., 1979). Following, the membrane was incubated in Blocking Buffer [5% (w/v) skimmed milk powder in Tris buffered-saline (TBS, pH 7.4) containing 0.1% Tween-20] overnight. For ligand blotting analysis (Toxin Overlay Assay - TOA), the membrane was incubated in 5 mL of TOA Blocking Buffer [3% (w/v) BSA in Tris buffered-saline (TBS, pH 7.4) containing 0.1% Tween-20] and containing WCT (Cry1Ac toxin) for 7 h at room temperature to interact with respective BBMV proteins. BSA was used as a negative control. The unbound toxins were removed by washing the blots with 5 mL of TOA Blocking Buffer, three times. Following, this blot was individually incubated with a polyclonal anti-*B. thuringiensis* Cry1Ab Toxin antibody (Abcam Inc.) (1:5,000 dilution in TOA Blocking Buffer) overnight; the section containing the activated Cry1Ac toxin was cut off from the membrane and incubated only with blocking buffer as a negative control. The membrane was then incubated with an anti-rabbit alkaline phosphatase-conjugated antibody (Sigma Aldrich). Finally, the blot was developed using NBT/BCIP (0.5 mM nitro blue tetrazolium, 0.57 mM 5-bromo-4-chloro-3-indolyl-phosphate) substrate in alkaline phosphatase buffer (10 mM Tris [pH 9,6], 100 mM NaCl, 5 mM MgCl_2_).

### 2.8 Sample preparation for LC-MS/MS analysis

Protein samples for mass spectrometry analysis were obtained from a 12% SDS-PAGE gel. Two bands corresponding to those that demonstrated binding to Cry1Ac in the ligand blot were excised from the gel. Coomassie R stain was removed from the excised bands. Reduction was performed by incubating the samples with 10 mM dithiothreitol (DTT) in 50 mM ammonium bicarbonate for 1 h at 30°C. Following reduction, alkylation was carried out by adding 40 mM iodoacetamide and incubating the samples for 30 min in the dark. After alkylation, the samples were washed with Milli-Q® water and dehydrated using 90% acetonitrile. The samples were then vacuum-dried to remove all traces of acetonitrile. Protein digestion was performed using commercial trypsin (Promega) at a 1:50 (w/w) enzyme-to-protein ratio. The samples were incubated with trypsin overnight at 37°C. Following digestion, 0.1% trifluoroacetic acid (TFA) was added to the samples, and they were incubated at room temperature for 1 h.

Tryptic peptides were purified using manual reversed-phase chromatography with Poros 50 R2 resin (PerSeptive Biosystems) and subsequently vacuum-dried. The peptides were then solubilized in 0.1% formic acid for injection into the mass spectrometer. Samples were analyzed using a nanoLC-MS/MS system consisting of an EASY II-nano LC (Proxeon Biosystem) coupled to a Q-Exactive Plus (Thermo Fisher Scientific) mass spectrometer. A total of 1 µg of peptides was loaded onto a manually packed pre-column (100 μm × 2 cm) containing C-18 ReproSil 5 μm resin (Dr. Maisch) and then separated on a 20 cm analytical column packed with Reprosil-pur C18-AQ 3 μm resin (Dr. Maisch). Chromatographic separation was performed using a linear gradient of solution B (95% ACN, 0.1% TFA) from 5% to 20 s, followed by an increase to 40% over 8 min, then to 95% over 4 min, and finally maintained at 95% for 8 min. The flow rate was set to 250 nL/min. Mass spectra % over 40 min were acquired in positive mode using a Top 10 data-dependent acquisition (DDA) method. MS1 scans were acquired in the Orbitrap analyzer with a mass range of 350–1,800 m/z, a resolution of 60,000 (at m/z 400), a minimum signal threshold of 10,000, and an isolation window of 2.0. The 10 most intense ions were selected for fragmentation by collision-induced dissociation (CID) with a normalized collision energy of 30 and a dynamic exclusion time of 30 s.

### 2.9 LC-MS/MS data analysis for protein identification

The spectra obtained from the LC-MS/MS analyses were processed using Proteome Discoverer 2.1 Software (Thermo Scientific) with the Sequest HT search engine against an *A. gemmatalis* protein database. This database was generated through a *de novo* assembly of the *A. gemmatalis* transcriptome based on raw data from a published study by Pezenti et al. (2021) (SRA accession number: PRJNA387150). For the search, the following parameters were used: precursor tolerance of 10 ppm, fragment tolerance of 0.1 Da, tryptic cleavage specificity, two maximum missed cleavage sites allowed, fixed modification of carbamidomethyl (Cys) and oxidation (Met). Peptides with high confidence were selected, and only identifications with q values equal to or less than 0.01 FDR were considered.

### 2.10 Gene expression analysis

Samples for analysis of gene expression were obtained in a feeding bioassay, in which a sublethal concentration of Bt spores (0.1323 mg/mL) was mixed in the artificial diet fed to 4th-5th instar larvae; the control group was fed in the artificial diet mixed with distilled water. After 24, 48 and 72 h of exposure to Bt, midguts were dissected and pooled (4 midguts per sample), with five biological replicates performed for the assay. Specimens were dissected and midgut epithelia were cleaned and used for total RNA extraction with TRI Reagent® (Sigma-Aldrich). Total RNA was quantified using NanoDrop One/One^c^ Microvolume UV-Vis Spectrophotometer (ThermoFisher Scientific), following the manufacturer’s recommendations. Total RNA (500 ng) was reverse transcribed using GoScript™ Reverse Transcriptase kit (Promega) following the manufacturer’s recommendations. For cDNA amplification, primers were designed following the re-annotation of a previously published *A. gemmatalis* transcriptome (Pezenti et al., 2021) (Table 1).

**TABLE 1:** Aminopeptidase N contigs from *A. gemmatalis* identified in the transcriptome (Pezenti et al., 2021).

Seq name^a^	Description^b^	Name^c^	Length^d^
TRINITY_DN21	ASU92546.1aminopeptidase N	AgAPN2	957
TRINITY_DN1045	QFP12817.1aminopeptidase N 1	AgAPN3	1,020
TRINITY_DN3057	AAL26894.1aminopeptidase N3	AgAPN4	954
TRINITY_DN2475	AWT22999.1aminopeptidase N5	AgAPN5	494
TRINITY_DN1607	ASU92547.1aminopeptidase N	AgAPN6	958
TRINITY_DN2592	AWT23001.1aminopeptidase N8	AgAPN8	938
TRINITY_DN465	XP_026737338.1aminopeptidase N	AgAPN10	939
TRINITY_DN4662	XP_047022343.1aminopeptidase N-like isoform X1	AgAPN11	1,092
TRINITY_DN37122	WAK99423.1aminopeptidase N 13		131
TRINITY_DN50751	XP_049705234.1aminopeptidase N isoform X2		174

^a^ Name of the contig in the transcriptome data;

^b^ Description of the contig identification through egg NOG-mapper (Cantalapiedra et al., 2021) in the transcriptome data;

^c^ All sequences were renamed after *in silico* analysis and those names are listed;

^d^ Length of the sequences in number of amino acids.

Primers were validated by RT-PCR using GoTaq Hot Start Colorless Master Mix (Promega) and the Veriti® 96-Well Thermal Cycler (Applied Biosystems). The cycling conditions were as follows: 94°C for 2 min, followed by 40 cycles of 94°C for 30 s, 52°C for 30 s, 72°C for 1 min. This stage was followed by 72°C for 10 min. Resulting amplification was visualized in 2% agarose gels using GelRed® Nucleic Acid Gel Stain (Biotium) following manufacturer’s recommendations.

Quantitative real-time PCR (qRT-PCR) was performed using qPCRBIO SyGreen Mix (PCR Biosystems) and the ViiA™ 7 Real-Time PCR System (Applied Biosystems). The qRT-PCR cycling conditions were as follows: 50°C for 2 min, 95°C for 10 min, followed by 40 cycles of 95°C for 30 s, 60°C for 30 s, and 72°C for 30 s. Relative expression levels were analyzed using the 2^−ΔΔCT^ method, using the expression level of the housekeeping gene elongation factor 1-alpha (ELF-1α) as a reference gene normalizer.

### 2.11 Statistical analysis

The results were submitted to one- and two-Way ANOVA (followed by Sidak’s multiple comparisons test), using the GraphPad Prism 8 software. Differences were considered significant at *p* < 0.05.

## 3 Results

### 3.1 Cry1Ac binds to epithelial cells in the midgut of *Anticarsia gemmatalis*

**FIGURE 1:**
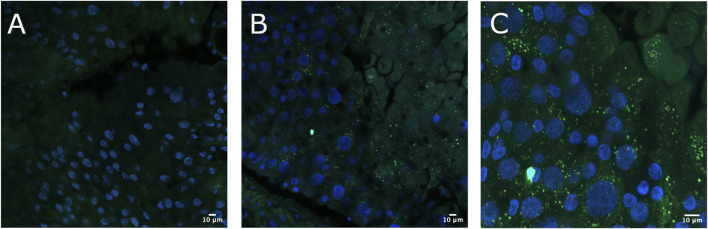
Immunofluorescence detection of Cry toxins in *A. gemmatalis* midgut tissues. Midgut samples were collected from larvae subjected to a feeding bioassay. The challenged group was fed an artificial diet containing *Bacillus thuringiensis* (Bt) spores at a concentration of 0.5 mg/mL, while the control group received only the artificial diet without spores. After 12 h of exposure, larval midguts were dissected, fixed, and incubated with an anti-*Bacillus thuringiensis* Cry1Ab Toxin antibody (1:250 dilution). Detection was performed using a confocal microscope following incubation with an Alexa Fluor 488-conjugated anti-rabbit secondary antibody. Nuclei were counterstained with DAPI for visualization. **(A)** Midgut from the control group showing no detectable signal from Cry antibodies at the brush border, included as a reference. **(B, C)** Detection of Cry toxins (green dots) at the apical brush border of the midgut epithelium using the Cry antibody. Images are representative of ten midguts.

To verify the immobilization of Cry toxins in the *A. gemmatalis* gut, we conducted an experiment where 5th instar *A. gemmatalis* larvae were fed with a sublethal Bt spore dose (0.5 mg/mL) for 12 h. After incubation with anti-*B. thuringiensis* Cry1Ab polyclonal antibody, Bt toxin was identified as a punctate pattern along the whole midgut epithelia (Figures 1B, C), suggesting a possible interaction with the apical portion of epithelial cells. Fluorescence was specific to the midgut and not observed in negative controls (Figure 1A). These findings suggest that Cry toxins interact with *A. gemmatalis*’ brush border membrane proteins, where APNs are known to be localized.

### 3.2 *In silico* analysis of aminopeptidases N from *Anticarsia gemmatalis*

To identify putative APN expressed by *A. gemmatalis*, we analyzed a previously published *A. gemmatalis* whole-body RNA-Seq dataset (Pezenti et al., 2021). The analysis of the *de novo* assembly of the transcriptome data resulted in the identification of 10 APN contigs, which are listed in Table 1. Of those, eight were full length sequences, hence being used in all posterior analysis; aminopeptidase N13 and aminopeptidase N-like isoform X2 were left out of most analyses since the sequences were only partial, impairing the results.

**FIGURE 2:**
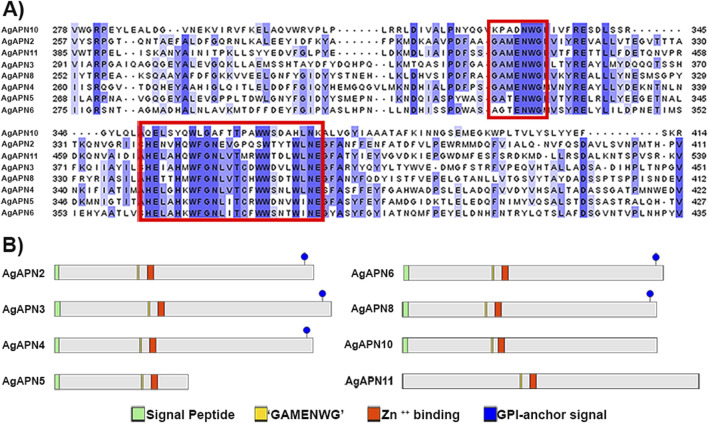
*In silico* identification and analysis of AgAPNs. AgAPN sequences were identified from a *de novo* assembled *A. gemmatalis* transcriptome and aligned against APN homologs. **(A)** Multiple sequence alignment of AgAPN sequences, partial. Red boxes indicate aminopeptidase motif “GAMENWG” and Zn^++^ binding motif “HEXXHX18E,” respectively. Alignment was conducted in Clustal Omega server, and the results were visualized as colored classification (percentage identity) using Jalview software (version 2.11.3.3). **(B)** Schematic representation of *A. gemmatalis*’ APNs. Green boxes correspond to the signal peptide; yellow boxes correspond to the aminopeptidase motif; red boxes correspond to the Zn^++^ binding motif; blue circles correspond to the GPI-anchor signal site.

Alignment of deduced translated amino acid sequences of AgAPNs through multiple sequence alignment and the search for the presence of characteristic gluzincin aminopeptidase activity motif “GAMENWG” and the consensus zinc-binding motif “HEXXHX18H” showed the presence of both motifs in nearly all sequences (Figure 2A).

The predicted N-terminal signal peptide cleavage site location was present in most of the sequences, varying between them; no signal peptide was predicted for AgAPN11 (Figure 2B). Further, a putative C-terminal GPI-anchor signal was predicted for AgAPN3, AgAPN4, AgAPN8, AgAPN6 and AgAPN2. No GPI-anchor site was predicted for AgAPN5, AgAPN10 and AgAPN11. Several potential O-glycosylation sites and one N-glycosylation site were identified in all sequences.

**FIGURE 3:**
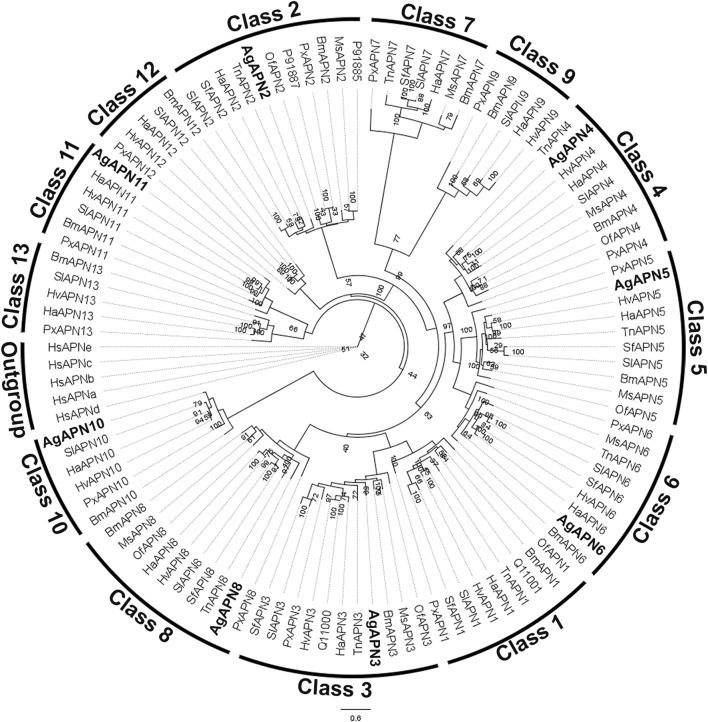
Phylogenetic tree of AgAPN sequences. Analysis conducted in RAxML software, using the maximum likelihood method with 1,000 bootstraps. Five *Homo sapiens*’ APNs were used as an outgroup and APNs from nine lepidopteran species (*Bombyx mori*, *Helicoverpa armigera*, *Heliothis virescens*, *Manduca sexta*, *Ostrinia furnacalis*, *Plutella xylostella*, *Spodoptera frugiperda*, *Spodoptera litura* and *Trichoplusia ni*) were used to conduct the analysis (Supplementary Table S1). Eigth APNs of *A. gemmatalis* were indicated by bold.

For the phylogenetic tree, APN proteins from different Lepidoptera species, belonging to the thirteen APN classes (Guo et al., 2020), were downloaded from NCBI and UniProt databases (Supplementary Table S1) to construct the tree using the maximum-likelihood method (Figure 3). This type of analysis has been broadly utilized to demonstrate the clustering of APN genes into different clusters, with high evolutionary conservation in each class (Guo et al., 2020; Wang et al., 2023). Following the genome-wide unified nomenclature and classification of APN genes in lepidopoteran insects (Guo et al., 2020), we designated APN genes in *A. gemmatalis* as AgAPN2, AgAPN3, AgAPN4, AgAPN5, AgAPN6, AgAPN8, AgAPN10 and AgAPN11.

### 3.3 Total aminopeptidase activity in the midgut

**FIGURE 4:**
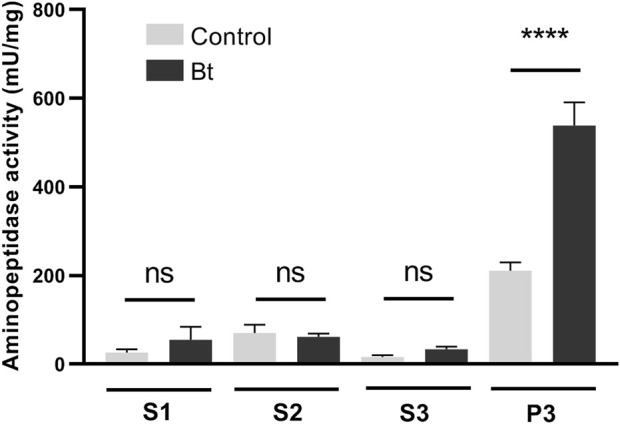
Total aminopeptidase N activity of BBMV preparation samples of *A. gemmatalis* larvae. 5th instar larvae were subjected to a sublethal concentration of Bt spores (0.1323 mg/mL) for 48 h, when midguts were dissected and subjected to the preparation of midgut brush border membrane vesicles protocol. Control groups were kept unchallenged. A higher aminopeptidase activity was expected in the BBMV fraction, where aminopeptidases N were expected to be detected. S1, supernatant 1, of the mild centrifugation; S2, supernatant 2, of the first ultracentrifugation; S3, supernatant 3, of the second ultracentrifugation; P3, pellet 3, of the second ultracentrifugation, ressuspended in HEPES. Bars represent the means and standard deviation of 3 biological replicates (pool of 4 midguts). Asterisks indicate significant difference (*P* < 0.0001, two-way ANOVA).

To evaluate aminopeptidase microvilli association and Bt modulation of aminopeptidase activity, we obtained fractionated samples from the midgut BBMVs of challenged and control individuals. Accordingly, we observed a Bt-upregulation of the aminopeptidase activity associated with the microvilli fraction (Figure 4).

### 3.4 Identification of Cry1Ac-binding proteins by LC-MS/MS

**FIGURE 5:**
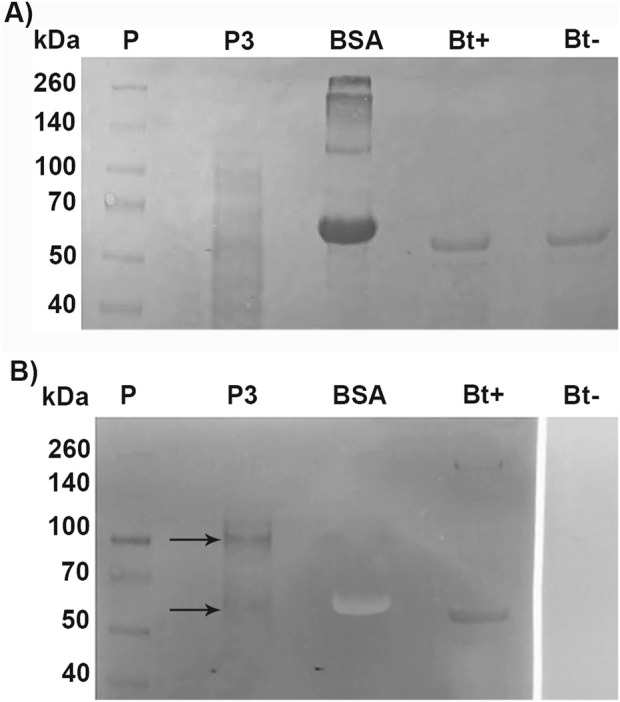
Toxin overlay assay of Cry1Ac toxin and BBMV proteins of *A. gemmatalis*. Midguts from 5th instar larvae were dissected (N = 200) and subjected to the preparation of midgut brush border membrane vesicles protocol. Proteins were separated by 12% SDS-PAGE and transferred to a nitrocellulose membrane; after blocking, the membrane was incubated with TOA Blocking Buffer containing activated Cry1Ac toxin, following incubation with Cry antibody. **(A)** Nitrocellulose membrane stained with Ponceau S; **(B)** membrane after blotting. Arrows indicate bands in P3 that present a positive result to the interaction with anti-Cry antibodies; Bt+ positively interacted with the antibody, as a positive control. P, protein standard; P3, pellet 3, of the second ultracentrifugation; BSA, bovine serum albumin (negative control); Bt+, activated Cry toxin (positive control); Bt−, activated Cry toxin not incubated with Cry antibody (negative control).

To identify Cry binding proteins in *A. gemmatalis*, we conducted a toxin overlay assay using Cry1Ac activated toxin on SDS-PAGE separated midgut BBMV proteins. The results presented in Figure 4 clearly show that Cry1Ac toxin interacted with membrane proteins, specifically with two bands of approximately 110-kDa and 65-kDa (Figure 5).

A new electrophoresis was performed from the same BBMV sample and bands in the same positions where there was binding to Cry1Ac in the TOA were excised and analyzed together by mass spectrometry. Results identified 1,152 proteins (Supplementary Table S1); the samples for this assay contained all proteins present in the two excised bands. Among them, seven AgAPNs were found (Table 2), suggesting that they could act as receptors for this toxin in *A. gemmatalis*’ midgut. The high number of unique peptides increases reliability in these results. It was possible to identify an alkaline phosphatase, which can also act as a receptor in the midgut, with a similar role in the progression of the infection.

**TABLE 2:** *A. gemmatalis*’ midgut proteins that demonstrated binding to Cry1Ac identified by mass spectrometry.

Description		Coverage (%)	PSMs	Peptides	Unique peptides	MW (kDa)
DN1045	Aminopeptidase N3	30.2	386	24	24	114.515
DN2592	Aminopeptidase N8	17.6	181	20	20	105.34
DN3057	Aminopeptidase N4	19.6	178	16	16	108.692
DN1607	Aminopeptidase N6	27.4	89	21	21	109.464
DN21	Aminopeptidase N2	21.4	81	16	16	108
DN2475	Aminopeptidase N5	18.2	49	9	7	55.881
DN4662	Aminopeptidase N-like isoform X1	2.28	3	2	2	123.359
DN56	Membrane-bound alkaline phosphatase-like	6	11	3	3	59.612

### 3.5 APN expression analysis after exposure to Bt spores

**FIGURE 6:**
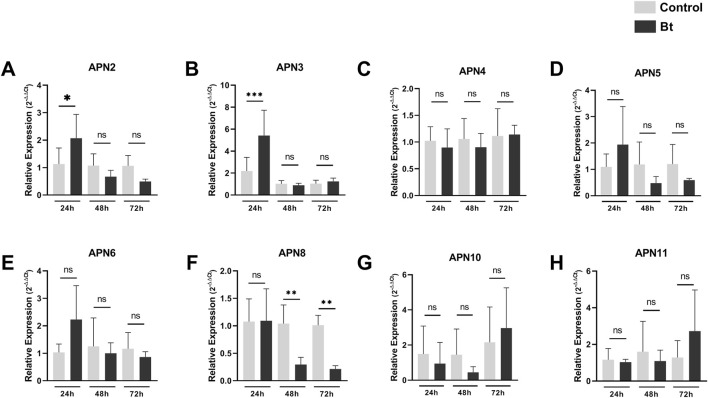
Expression analysis of AgAPNs in *A. gemmatalis* larvae midguts. Samples were obtained from midguts of 5th instar larvae subjected to a sublethal concentration of Bt spores in the challenged group, replaced by water in the control group. Midguts were dissected after 24, 48 and 72 h of exposure, following RNA extraction. Each graph shows the relative expression levels of AgAPNs in the midgut of *A. gemmatalis*’ larvae in each condition. **(A)** AgAPN2, **(B)** AgAPN3, **(C)** AgAPN4, **(D)** AgAPN5, **(E)** AgAPN6, **(F)** AgAPN8, **(G)** AgAPN10, **(H)** AgAPN11. Relative expression calculated through the 2^−ΔΔCt^ method using ELF-1α as a reference gene. Gray bars correspond to control individuals and black bars correspond to challenged individuals. The analysis was conducted with five biological replicates, each consisting in a pool of four midguts, and two technical replicates. Asterisks indicate significant difference (^*^ *p* < 0.02; ^**^ *p* < 0.005; ^***^ *p* < 0.0005, one-way ANOVA).

To evaluate differences in expression of APNs between control individuals and challenged with Bt spores individuals, we performed RT-qPCR analysis of all AgAPNs described in this work upon 24, 48 and 72 h of exposure (Figure 6). Regarding the different conditions of exposure, AgAPN10, AgAPN4, AgAPN5, AgAPN6 and AgAPN11 did not present any significant difference in expression between the challenged and control groups in any of the time points. Genes of AgAPN2 and AgAPN3 of the challenged group presented an upregulation in 24 h of exposure in comparison with the control group, with a decrease in expression in the following time points and no significant difference from the control group. AgAPN8, on the other hand, presented downregulation of expression from 48 to 72 h in comparison with the control group. AgAPN5 and AgAPN6 did not present any significant difference of expression between treatments in each time point but did present a tendency downregulation in the challenged group from 48 to 72 h in relation to the initial period of exposure.

## 4 Discussion

*B. thuringiensis* (Bt) is the most widely used bacteria for control of insect pests due to its production of parasporal entomopathogenic crystals during sporulation, known as Cry toxins (Liu et al., 2021). These are toxic by ingestion, acting in the midgut of susceptible insects where, after solubilization and activation, the Cry proteins oligomerize and form pores, which are then inserted in the epithelial membrane. This disrupts the cellular membrane, causing leakage of cellular contents and osmotic imbalance and allowing gut bacteria to invade the cells and the hemolymph, leading to septicemia; this ultimately triggers the infected insect to stop eating, alongside with the infection events that culminates in insect death (Liu et al., 2021). This infection triggers host responses, modulating different pathways and molecules, to counteract these effects (Pinos et al., 2021).

The binding of Cry1Ac toxin to brush border membrane vesicles (BBMV) from *A. gemmatalis* midguts was demonstrated by the fractionation of total aminopeptidase activity, revealing increased activity in BBMV fractions of larvae fed a diet containing Bt spores. This rise in aminopeptidase activity is consistent with previous reports that describe elevated APN activity following Cry toxin exposure (Ingle et al., 2001). These findings were further supported by immunohistochemistry, which confirmed the interaction of Cry1Ac with the midgut epithelium, as previously shown in other lepidopterans (Chauhan et al., 2021; Valaitis, 2011; Chen et al., 2005; Aimanova, Zhuang, and Gill, 2006).

Despite its importance as a major soybean defoliator, few studies addressed the molecular basis of Cry toxins’ mode of action on the midgut of *A. gemmatalis*. Bel et al. (2017), Bel et al. (2019) and Fiuza et al. (2013) identified Cry toxin binding to the midgut brush border tissue but did not identify a functional receptor for these toxins. Da Silva et al. (2019) characterized a membrane-associated alkaline phosphatase that binds to Cry1Ac toxin, but further studies would be required to describe it as a functional receptor. APNs have been extensively described as Cry toxin receptors in many insect pest species; these proteins consist in a group of glycoproteins attached to the midgut epithelial membrane through a GPI-anchor (Sato, 2002; Pardo-Lopez et al., 2013; Flores-Escobar et al., 2013; Herrero et al., 2016; Liu et al., 2021).

Ten APNs were identified from the transcriptome data of *A. gemmatalis* (Pezenti et al., 2021) with eight full-length sequences that were subsequently analyzed. A phylogenetic tree containing APNs from seven other lepidopteran species, belonging to the eight described classes of APNs in insects (Crava et al., 2010; Hughes, 2014; Lin et al., 2014), showed that the eight AgAPNs were distributed into different classes and named accordingly (AgAPN2, AgAPN3, AgAPN4, AgAPN5, AgAPN6, AgAPN8, AgAPN10 and AgAPN11), following the genome-wide unified nomenclature and classification of APN genes in lepidopteran insects (Guo et al., 2020). *In silico* analysis of the sequences revealed the presence of characteristic motifs “GAMENWG” and “HEXXHX18E” in most of the sequences; AgAPN10, AgAPN5 and AgAPN6 do not present the entire “GAMENWG” motif and AgAPN10 also does not present the entire “HEXXHX18E” motif. C-terminal GPI-anchor signal was identified for AgAPN2, AgAPN3, AgAPN4, AgAPN6 and AgAPN8 and N-terminal signal peptide cleavage site was identified in all sequences except for AgAPN11. It was interesting to note that AgAPN11 lacked both C-terminal GPI-anchor signal and N-terminal signal peptide cleavage site; a similar occurrence has been described for AjAPN9 in *Achaea janata* (Chauhan et al., 2021).

APN was initially shown as a Cry1Ac binding protein through TOA ligand blot analysis (Knight et al., 1994; Sangadala et al., 1994). Our present ligand-binding study revealed interaction of *A. gemmatalis* midgut epithelial cell membrane proteins with the activated Cry1Ac toxin obtained from *B. thuringiensis* sor. *kurstaki*, with a prominent interaction primarily seen with a ∼100 kDa protein, as well as a minor interaction with a ∼60 kDa protein. Corresponding bands from an SDS-PAGE were then excised and the identities of the proteins were confirmed by mass spectrometry analysis, in which several AgAPN sequences were identified; the same experiment was conducted for *M. sexta*, in which mass spectrometry of a band that positively bound to Cry2Ab toxin identified the protein as an APN (Onofre et al., 2017), and for *Athethis lepigone*, another lepidopteran species (Wang et al., 2017). Of the seven AgAPNs identified, four figured among the 100 highest PSMs (peptide spectrum match), which corresponds to a score of matches between experimental MS/MS spectra to the theoretical spectra predicted for the tryptic peptides, in which proteins with peptides best matched with the experimental spectrum are considered the most likely candidates (Wu et al., 2006). Therefore, supporting the presence of those proteins in the samples analyzed. An alkaline phosphatase (ALP), present in the transcriptome data, was also identified in the mass spectrometry analysis; this protein is frequently grouped with APNs, mainly because it is also anchored to the membrane by an GPI-anchor, besides displaying a similar role during the toxin’s mode of action (Ziga-Navarrete et al., 2021; Liu et al., 2021). As mentioned before, our group published a work characterizing an ALP from *A. gemmatalis* and describing an *in vitro* binding to Cry1Ac (da Silva et al., 2019); our data corroborate this finding.

In resistant strains, expression of genes encoding Cry receptors is commonly downregulated, whereas non-receptor paralogs are upregulated, believed to compensate for the absence of the down-regulated ones (Guo et al., 2022). The cabbage looper, *Trichloplusia ni*, presented down-regulation of TnAPN1 in Cry-resistant strains (Tiewsiri and Wang, 2011); tolerant strains of the castor semilooper larvae (*A. janata*) presented reduced expression of AjAPN2 while AjAPN4 was up-regulated (Chauhan et al., 2021); resistant strains of *Spodoptera exigua* reared in-lab lacked expression of a SeAPN1, whereas susceptible insects positively expressed this gene (Herrero et al., 2005). For *Chilo suppressalis*, knockdown of CsAPN6 and CsAPN8 reduced the larvae’s sensitivity to transgenic rice expressing Cry1 toxins (Sun et al., 2020), and previous studies had demonstrated the involvement of APNs in the toxin’s mode of action (Qiu et al., 2017; Wang et al., 2017). In our analysis we could identify an upregulation of AgAPN2 and AgAPN3 genes at 24 h of exposure to Bt spores, followed by a decrease in the following time points, as well as AgAPN5 and AgAPN6. Expression levels of AgAPN8 also demonstrated a downregulation, with great significance values. All these results could indicate a possible role of some AgAPNs in the mode of action of the Cry1Ac toxin in *A. gemmatalis*, but further studies are required for their characterization as putative receptors in the midgut, such as silencing through RNA interference and CRISPR/Cas9.

In conclusion, the identification of some AgAPNs as Cry-binding proteins in the ligand blot, as well as the expression analysis of all isoforms, could give us a direction in the sense of which of those proteins could be in fact involved in this process in *A. gemmatalis* midgut. Overall, this study gives a perspective of Cry1Ac possible receptors in *A. gemmatalis*, enhancing our understanding of this mode of action in this great agricultural pest.

## Data Availability

The mass spectrometry proteomics data have been deposited to the ProteomeXchange Consortium via the PRIDE (Perez-Riverol et al., 2022) partner repository with the dataset identifier PXD055041.
