# Supplementary material for: Cry1Ac toxin binding in the velvetbean caterpillar Anticarsia gemmatalis: study of midgut aminopeptidases N
Source: Front Physiol. 2024 Oct 29;15:1484489. doi: 10.3389/fphys.2024.1484489 (PMC11554492; doi:10.3389/fphys.2024.1484489)
Supplement: Supplementary file 2 [file Table1.DOCX]

**Supplementary Table 1: APN sequences utilized in the phylogenetic analysis.**

| **Name** | **Species** | **Family** | **Source** | **Acession number** | **Size (aa)** |
| --- | --- | --- | --- | --- | --- |
| BmAPN1 | *Bombyx mori* | Bombycidae | NCBI | NP_001037013.1 | 986 |
| BmAPN2 | *Bombyx mori* | Bombycidae | NCBI | NP_001036834.1 | 948 |
| BmAPN3 | *Bombyx mori* | Bombycidae | NCBI | NP_001104835.1 | 1005 |
| BmAPN4 | *Bombyx mori* | Bombycidae | NCBI | XP_012552709.1 | 949 |
| BmAPN5 | *Bombyx mori* | Bombycidae | NCBI | AFK85018.1 | 945 |
| BmAPN6 | *Bombyx mori* | Bombycidae | NCBI | AFK85019.1 | 881 |
| BmAPN7 | *Bombyx mori* | Bombycidae | NCBI | NP_001269149.1 | 855 |
| BmAPN8 | *Bombyx mori* | Bombycidae | NCBI | XP_062525938.1 | 772 |
| BmAPN9 | *Bombyx mori* | Bombycidae | NCBI | NP_001266329 | 987 |
| BmAPN10 | *Bombyx mori* | Bombycidae | NCBI | NP_001269273 | 941 |
| BmAPN11 | *Bombyx mori* | Bombycidae | NCBI | XP_012552714 | 1095 |
| BmAPN12 | *Bombyx mori* | Bombycidae | NCBI | XP_004922953 | 1024 |
| BmAPN13 | *Bombyx mori* | Bombycidae | NCBI | NP_001296554 | 936 |
| HaAPN1 | *Helicoverpa armigera* | Noctuidae | NCBI | XP_021192756.1 | 1014 |
| HaAPN2 | *Helicoverpa armigera* | Noctuidae | NCBI | XP_021192782.1 | 941 |
| HaAPN3 | *Helicoverpa armigera* | Noctuidae | NCBI | XP_021192748.1 | 1013 |
| HaAPN4 | *Helicoverpa armigera* | Noctuidae | NCBI | XP_021192757.3 | 951 |
| HaAPN5 | *Helicoverpa armigera* | Noctuidae | NCBI | XP_021192755.3 | 1047 |
| HaAPN6 | *Helicoverpa armigera* | Noctuidae | NCBI | XP_021192759.3 | 961 |
| HaAPN7 | *Helicoverpa armigera* | Noctuidae | NCBI | EU328182.1 | 742 |
| HaAPN8 | *Helicoverpa armigera* | Noctuidae | NCBI | XP_063893399.1 | 939 |
| HaAPN9 | *Helicoverpa armigera* | Noctuidae | NCBI | XP_021192786 | 985 |
| HaAPN10 | *Helicoverpa armigera* | Noctuidae | NCBI | XP_021197747 | 942 |
| HaAPN11 | *Helicoverpa armigera* | Noctuidae | NCBI | XP_021197746 | 1066 |
| HaAPN12 | *Helicoverpa armigera* | Noctuidae | NCBI | XP_021192750 | 1003 |
| HaAPN13 | *Helicoverpa armigera* | Noctuidae | NCBI | XP_021190039 | 974 |
| HvAPN1 | *Heliothis virescens* | Noctuidae | NCBI | AAF08254 | 1010 |
| HvAPN3 | *Heliothis virescens* | Noctuidae | NCBI | AAC46929 | 1009 |
| HvAPN4 | *Heliothis virescens* | Noctuidae | NCBI | AAK58066 | 950 |
| HvAPN5 | *Heliothis virescens* | Noctuidae | NCBI | PCG64448 | 1047 |
| HvAPN6 | *Heliothis virescens* | Noctuidae | NCBI | PCG68031 | 963 |
| HvAPN8 | *Heliothis virescens* | Noctuidae | NCBI | PCG64451 | 936 |
| HvAPN9 | *Heliothis virescens* | Noctuidae | NCBI | PCG66277 | 971 |
| HvAPN10 | *Heliothis virescens* | Noctuidae | NCBI | PCG70717 | 937 |
| HvAPN11 | *Heliothis virescens* | Noctuidae | NCBI | PCG70716 | 1048 |
| HvAPN12 | *Heliothis virescens* | Noctuidae | NCBI | PCG65583 | 989 |
| HvAPN13 | *Heliothis virescens* | Noctuidae | NCBI | PCG74619 | 1405 |
| MsAPN2 | *Manduca sexta* | Sphingidae | NCBI | XP_030032717.1 | 942 |
| MsAPN3 | *Manduca sexta* | Sphingidae | NCBI | XP_030032724.2 | 1010 |
| MsAPN4 | *Manduca sexta* | Sphingidae | NCBI | KAG6458920.1 | 947 |
| MsAPN5 | *Manduca sexta* | Sphingidae | NCBI | KAG6458913.1 | 947 |
| MsAPN6 | *Manduca sexta* | Sphingidae | NCBI | XP_030032729.2 | 954 |
| MsAPN7 | *Manduca sexta* | Sphingidae | NCBI | XP_030032734.1 | 855 |
| MsAPN8 | *Manduca sexta* | Sphingidae | NCBI | XP_030032733.2 | 927 |
| OfAPN1 | *Ostrinia furnacalis* | Crambidae | NCBI | ACX85726.2 | 994 |
| OfAPN2 | *Ostrinia furnacalis* | Crambidae | NCBI | XP_028172227.1 | 940 |
| OfAPN3 | *Ostrinia furnacalis* | Crambidae | NCBI | XP_028172113.1 | 1014 |
| OfAPN4 | *Ostrinia furnacalis* | Crambidae | NCBI | ACF34998.2 | 951 |
| OfAPN5 | *Ostrinia furnacalis* | Crambidae | NCBI | XP_028172430.1 | 953 |
| OfAPN8 | *Ostrinia furnacalis* | Crambidae | NCBI | XP_028172441.1 | 925 |
| PxAPN1 | *Plutella xylostella* | Plutellidae | NCBI | NP_001292446.1 | 988 |
| PxAPN2 | *Plutella xylostella* | Plutellidae | NCBI | NP_001296022.1 | 946 |
| PxAPN3 | *Plutella xylostella* | Plutellidae | NCBI | AAF01259.2 | 942 |
| PxAPN4 | *Plutella xylostella* | Plutellidae | NCBI | XP_011560697.3 | 957 |
| PxAPN5 | *Plutella xylostella* | Plutellidae | NCBI | ADD39718.1 | 950 |
| PxAPN6 | *Plutella xylostella* | Plutellidae | NCBI | XP_037966916.2 | 959 |
| PxAPN7 | *Plutella xylostella* | Plutellidae | NCBI | QBI71862.1 | 854 |
| PxAPN8 | *Plutella xylostella* | Plutellidae | NCBI | QBI71863.1 | 928 |
| PxAPN9 | *Plutella xylostella* | Plutellidae | NCBI | MG873056 | 1004 |
| PxAPN10 | *Plutella xylostella* | Plutellidae | NCBI | MG873057 | 934 |
| PxAPN11 | *Plutella xylostella* | Plutellidae | NCBI | MG873058 | 1121 |
| PxAPN12 | *Plutella xylostella* | Plutellidae | NCBI | MG873059 | 999 |
| PxAPN13 | *Plutella xylostella* | Plutellidae | NCBI | MG873060 | 1007 |
| SfAPN1 | *Spodoptera frugiperda* | Noctuidae | NCBI | XP_050562657.1 | 1020 |
| SfAPN2 | *Spodoptera frugiperda* | Noctuidae | NCBI | WAK99412.1 | 970 |
| SfAPN3 | *Spodoptera frugiperda* | Noctuidae | NCBI | XP_035438967.1 | 993 |
| SfAPN5 | *Spodoptera frugiperda* | Noctuidae | NCBI | XP_035438688.2 | 984 |
| SfAPN6 | *Spodoptera frugiperda* | Noctuidae | NCBI | XP_035438719.2 | 956 |
| SfAPN7 | *Spodoptera frugiperda* | Noctuidae | NCBI | WAK99417.1 | 860 |
| SfAPN8 | *Spodoptera frugiperda* | Noctuidae | NCBI | WAK99418.1 | 936 |
| SlAPN1 | *Spodoptera litura* | Noctuidae | NCBI | XP_22835594.1 | 1000 |
| SlAPN2 | *Spodoptera litura* | Noctuidae | NCBI | XP_022825009.1 | 974 |
| SlAPN3 | *Spodoptera litura* | Noctuidae | NCBI | XP_022834840.1 | 1011 |
| SlAPN4 | *Spodoptera litura* | Noctuidae | NCBI | XP_022825014.1 | 856 |
| SlAPN5 | *Spodoptera litura* | Noctuidae | NCBI | XP_022825006.1 | 1005 |
| SlAPN6 | *Spodoptera litura* | Noctuidae | NCBI | XP_022825010.1 | 953 |
| SlAPN7 | *Spodoptera litura* | Noctuidae | NCBI | XP_022825013.1 | 862 |
| SlAPN8 | *Spodoptera litura* | Noctuidae | NCBI | ABN04204.1 | 766 |
| SlAPN9 | *Spodoptera litura* | Noctuidae | NCBI | XP_022825007 | 978 |
| SlAPN10 | *Spodoptera litura* | Noctuidae | NCBI | XP_022825011 | 938 |
| SlAPN11 | *Spodoptera litura* | Noctuidae | NCBI | XP_022825002 | 1099 |
| SlAPN12 | *Spodoptera litura* | Noctuidae | NCBI | XP_022834841 | 1005 |
| SlAPN13 | *Spodoptera litura* | Noctuidae | NCBI | XP_022827407 | 966 |
| TnAPN1 | *Trichoplusia ni* | Noctuidae | NCBI | AAX39863.1 | 982 |
| TnAPN2 | *Trichoplusia ni* | Noctuidae | NCBI | AAX39864.1 | 939 |
| TnAPN3 | *Trichoplusia ni* | Noctuidae | NCBI | AAX39865.1 | 1011 |
| TnAPN4 | *Trichoplusia ni* | Noctuidae | NCBI | AAX39866.1 | 948 |
| TnAPN5 | *Trichoplusia ni* | Noctuidae | NCBI | AEA29693.1 | 940 |
| TnAPN6 | *Trichoplusia ni* | Noctuidae | NCBI | AEA29694.1 | 959 |
| TnAPN7 | *Trichoplusia ni* | Noctuidae | NCBI | XP_026737165.1 | 860 |
| TnAPN8 | *Trichoplusia ni* | Noctuidae | NCBI | XP_026737407.1 | 933 |
| HsAPNa | *Homo sapiens* | Hominidae | NCBI | NP_001141.2 | 967 |
| HsAPNb | *Homo sapiens* | Hominidae | NCBI | BAD93155.1 | 977 |
| HsAPNc | *Homo sapiens* | Hominidae | NCBI | AAA51719.1 | 967 |
| HsAPNd | *Homo sapiens* | Hominidae | NCBI | KAI4059404.1 | 967 |
| HsAPNe | *Homo sapiens* | Hominidae | NCBI | CAA31640.1 | 967 |
| P91887 | *Plutella xylostella* | Plutellidae | UniProt | P91887 | 946 |
| P91885 | *Manduca sexta* | Sphingidae | UniProt | P91885 | 942 |
| Q11001 | *Manduca sexta* | Sphingidae | UniProt | Q11001 | 990 |
| Q11000 | *Heliothis virescens* | Noctuidae | UniProt | Q11000 | 1009 |
